# Functionalization of Metal and Carbon Nanoparticles with Potential in Cancer Theranostics

**DOI:** 10.3390/molecules26113085

**Published:** 2021-05-21

**Authors:** Nicolò Mauro, Mara Andrea Utzeri, Paola Varvarà, Gennara Cavallaro

**Affiliations:** 1 Lab of Biocompatible Polymers, Department of Biological, Chemical and Pharmaceutical Sciences and Technologies (STEBICEF), University of Palermo, via Archirafi 32, 90123 Palermo, Italy; maraandrea.utzeri@unipa.it (M.A.U.); paola.varvara@unipa.it (P.V.); gennara.cavallaro@unipa.it (G.C.); 2 Advanced Technologies Network Center, University of Palermo, Viale delle Scienze, Ed. 18, 90128 Palermo, Italy

**Keywords:** cancer therapy, diagnosis, carbon nanoparticles, carbon dots, graphene, biopolymers, conjugation, precision medicine, theranostics

## Abstract

Cancer theranostics is a new concept of medical approach that attempts to combine in a unique nanoplatform diagnosis, monitoring and therapy so as to provide eradication of a solid tumor in a non-invasive fashion. There are many available solutions to tackle cancer using theranostic agents such as photothermal therapy (PTT) and photodynamic therapy (PDT) under the guidance of imaging techniques (e.g., magnetic resonance—MRI, photoacoustic—PA or computed tomography—CT imaging). Additionally, there are several potential theranostic nanoplatforms able to combine diagnosis and therapy at once, such as gold nanoparticles (GNPs), graphene oxide (GO), superparamagnetic iron oxide nanoparticles (SPIONs) and carbon nanodots (CDs). Currently, surface functionalization of these nanoplatforms is an extremely useful protocol for effectively tuning their structures, interface features and physicochemical properties. This approach is much more reliable and amenable to fine adjustment, reaching both physicochemical and regulatory requirements as a function of the specific field of application. Here, we summarize and compare the most promising metal- and carbon-based theranostic tools reported as potential candidates in precision cancer theranostics. We focused our review on the latest developments in surface functionalization strategies for these nanosystems, or hybrid nanocomposites consisting of their combination, and discuss their main characteristics and potential applications in precision cancer medicine.

## 1. Introduction

**Figure 1:**
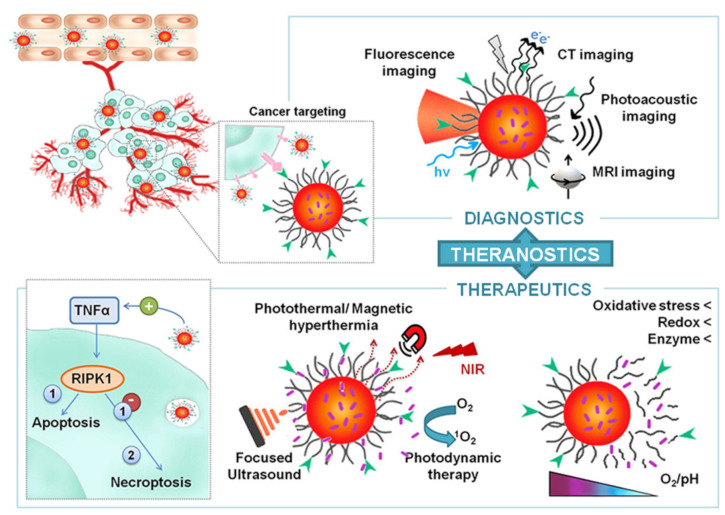
Conceptual representation of the term theranostics. Nanoparticles with theranostic properties are designed to be accumulated into the target site (tumor) in order to image, monitor and treat the disease. Different therapeutic and diagnostic approaches can be combined to have a multimodal clinical diagnosis and precise and personalized treatments.

Cancer theranostics is an emerging field of nanotechnology that combines therapy and diagnosis in a single smart tool, providing real-time monitoring of treatment progress and efficacy [1,2]. The potential of this multimodal approach is manifold as it offers to simultaneously achieve efficient treatments with an immediate imaging feedback, opening the way to the implementation of highly targeted and innovative patient-tailored therapies that can be customized according to the individual therapeutic response [3,4,5]. To fulfill this purpose, several theranostic materials were explored to date, including metallic nanoparticles (e.g., silver, zinc, gold and iron oxide nanoparticles) [6,7,8,9,10,11,12,13,14,15], carbon nanomaterials (e.g., nanotubes, fullerenes, nanodots and graphene) [16,17,18,19,20,21,22,23,24,25], rare-earth elements-based structures [26,27] and polymeric assemblies [28,29,30,31]. Even though unified by the same goal, theranostic nanomaterials can avail of different features that are direct effects of their physical–chemical, optoelectronic, and magnetic properties, as well as the result of the accurate design of the intelligent nanodevice (Figure 1).

Among the most promising theranostic tools proposed in recent literature panorama, gold nanoparticles (GNPs), superparamagnetic iron oxide nanoparticles (SPIONs), carbon nanodots (CDs) and graphene oxide (GO) have shown intriguing characteristics exploitable in biomedicine and especially in cancer theranostics.

GNPs are a varied class of colloidal gold with a controllable shape and characteristics [32,33]. As a noble metal, gold shows physical–chemical plasmonic properties that are crucial for their application in cancer theranostics, as they are responsible for both imaging and therapeutic features. For these reasons, GNPs were largely employed to design smart biosensors as contrast agents (e.g., in computed tomography-CT and photoacoustic imaging-PA), as well as for therapeutic purposes in image-guided photothermal therapy (IG-PTT) [34].

SPIONs have been explored as efficient contrast agents in cancer magnetic resonance imaging (MRI) combined to chemo- and magnetotherapy. The viewpoint is that SPIONs can be accumulated into the tumor mass through magnetic targeting under the guidance of external static magnetic fields, and simultaneously used to release anti-cancer drugs and heat by using alternated magnetic fields directly in the site of action. However, the MRI efficacy of SPIONs remains much lower if compared with Gd-based drugs (the gold standard in clinics), and further efforts should be made to overcome these limitations [35,36].

To date, carbon-based nanomaterials such as GO and CDs have attracted considerable attention, since they are cost-effective, stable, biodegradable and possess excellent photothermal conversion in the near infrared (NIR) region, which make them potential candidates as nanoheaters useful in photoacoustic imaging and PTT applications. In particular, nano-GO has been exploited for biosensing applications, and to simultaneously release local heat and photothermal-induced release of huge amounts of anticancer drugs under the guidance of PA. However, their batch-to-batch size and structural characteristics are hardly controllable and thus a real-world medical application for GO is difficult. On the contrary, CDs are emerging 0-D nanomaterials with established size and surface functionalization, endowed with a smart combination of optical (high NIR PT conversion, high fluorescence quantum yield in the red-NIR region) and biological (bioeliminable, biodegradable and biocompatible) properties, that seem to overcome the GO-related drawbacks, thus paving the way for future studies to develop promising theranostic agents for precision cancer therapy and diagnosis [37,38,39].

Although there are many examples of surface functionalization of nanomaterials for cancer theranostic applications, the proposed approaches are quite complex, and mainly yield to heterogeneous nanoplatforms whose industrial production for a real-world use is precluded. Considering the high-level of complexity as well as the difficulty to obtain properties with a suitable batch to batch consistency, this kind of nanotool usually do not meet the regulatory and translational criteria for commercialization. On the contrary, the application of carefully controlled synthesis and functionalization procedures may lead to highly ordered architectures exhibiting the desired properties in an extremely reproducible fashion. Bearing in mind that theranostic nanostructure features are typically tailorable with a high grade of precision during the synthesis stage, the uniformity of the starting material plays a critical role, the reason why bottom-up approaches are generally preferred addressing the production of most reliable tools, and are concurrently more suitable for translational theranostic research [40,41,42].

In this context, this review displays a comparative overview of the current state of the art, examining the characteristics and potentials of some of the most promising classes of theranostic nanomaterials, aiming to give a panoramic view that might be helpful for the researcher approaching the study of functionalized theranostic nanotools in cancer precision medicine.

## 2. Synthesis and Properties of Nano-sized Particles with Theranostic Properties

### 2.1. Gold Nanoparticles (GNPs)

**Figure 2:**
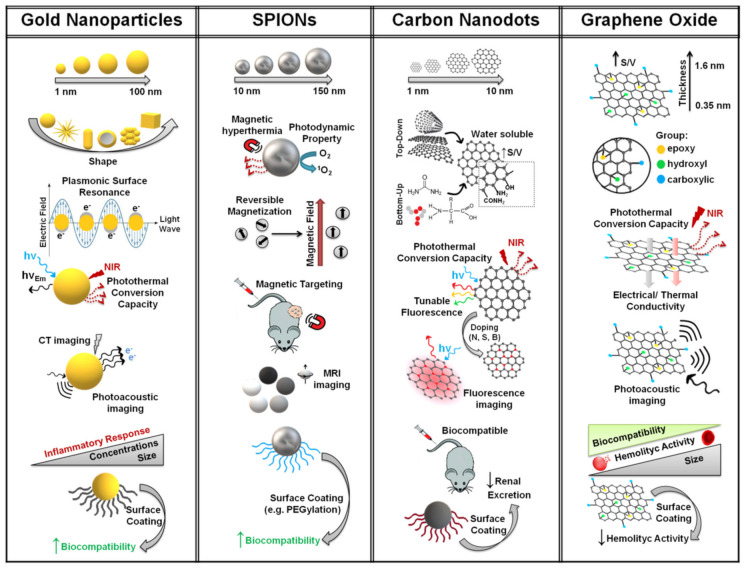
Schematic representation of the main properties and features of metal and carbon nanoparticles used in cancer theranostics. The structure and therapeutic/diagnostic potential is reported according to the literature panorama.

Noble metal nanoparticles, among which the main family is represented by gold nanoparticles (GNPs), have fascinated researchers in the field of nanomedicine for decades and are being extensively used owing to their well-established biocompatibility and physicochemical properties, which warrant their use in medical applications (Figure 2) [15,43,44].

Among all, GNPs are considered as potential candidates for clinical applications in cancer theranostics due to their chemical inertness, the unique optoelectronic properties such as Raman scattering, fluorescence and surface plasmonic resonance (SPR), and the high versatility in terms of surface engineering, thus leading to nanosystems with tunable biological properties (e.g., high biocompatibility and selective accumulation towards tissues) [45,46]. SPR is of particular interest since when GNPs are exposed to light corresponding to the plasmonic band, the oscillating electromagnetic field of the light source provokes a coherent oscillation of the free surface electron, which induces a charge separation with respect to the lattice, thus implying a formation of an oscillating dipole aligned with the electric field of the light called plasmon surface resonance [47]. SPR is highly affected by GNPs dimension, shape and concentration, and enhances radiative properties such as absorption and scattering, offering a huge choice of applications in biological and medical fields [48]. For instance, GNPs have been proven to be efficient photothermal nanosystems able to kill cancer cells and reduce tumor mass after a biocompatible near-infrared (NIR) laser exposure (photothermal therapy—PTT) [12,49]. GNPs, especially nanorods of 30–100 nm in length and aspect ratio smaller than 5, can indeed strongly absorb light in the range of 700–1000 nm (NIR light) as the result of the SPR and convert it into heat by the fast electron–phonon/phonon–phonon processes, which makes gold nanorods a useful candidate for cancer photothermal therapy [50]. Based on the combination of the EPR effect and active targeting, GNPs can precisely reach tumor cells in the body, thus they can be used as molecular lancets after exposure to the appropriate laser light so as to target only the tumor mass [51,52]. In addition, thanks to their optical properties (i.e., fluorescence, X-ray computed tomography—CT) GNPs can be also employed as powerful tools in image-guided photothermal therapy (IG-PTT) to tackle solid tumors and the outbreak of metastasis [53].

The numerous varieties of GNPs comprise nanospheres (GNSs), nanorods (GNRs), nanoshells (GNShs), nanocubes (GNCs), nanostars (GNSts), nanocages (GNCgs), nanobipyramids (GNBPys) and many other simple or composite structures [32,33]. It is no coincidence that their physicochemical characteristics are reflected in different optical properties that can be exploited in diverse applications in the biomedical field. In this context, one of the strongest points of this class of nanoparticles is the precision with which their optoelectronic properties can be tailored during synthesis, conveying towards the desired application. Among the best approaches to achieve high control of the shape and size of gold nanoparticles, there are certainly bottom-up approaches. As much as these include electrochemical methods [54,55], template methods [56,57] and seed-mediated methods, this latter has achieved great success due to its simplicity, high yields, and ease of accurately controlling the size and aspect ratio of GNPs produced by modulation of key parameters [58,59,60]. The seed-mediated method is mainly divided into two synthetic steps that provide, respectively, the nucleation of spheroidal structures of a few nanometers of diameter, and their growth via gold deposition on newly formed nuclei in a reducing environment. In this way, it is generally possible to control with extreme precision the shape and size of gold nanoparticles, simply by carefully adjusting scalable parameters such as temperature, reagent concentrations and the use of additives that boost growth in preferential directions, sculpturing GNPs with the most varied architectures and aspect ratios [61]. It is noteworthy that the high shape and size variability of this particular class of nanomaterial do not allow a simple correlation between their physicochemical properties and biocompatibility. As a rule, GNPs are not biodegradable and have a diameter higher than 10 nm (bigger than the renal excretion cutoff). As consequence, they have the tendency to be accumulated in high perfused organs (liver, intestine, kidney and spleen) after the administration in vivo. Only ultra-small GNPs with diameter lower than 5 nm can be directly eliminated after in vivo administration, but unfortunately induce greater deleterious effects as supported by the DNA damage and their location inside the cell nucleus [62]. Besides, ultra-small GNPs show the highest widespread and crossing the blood–brain barrier.

Despite GNPs have a huge potential in the biomedical field, the lyophobic nature of these colloids requires a hydrophilic/amphiphilic decoration that can prevent aggregation and increase biocompatibility to enable in vivo application [63]. For this reason, many surface agents such as small molecules or polymers have been to date used to ensure their stability in aqueous media, mostly exploiting the sulfur-gold chemistry, the presence of amino groups or other types of interaction with the gold surface [64,65,66,67,68].

The versatility of the synthetic procedures of GNPs, along with the possibility to tailor the optical properties, makes GNPs useful nanomaterials for a plethora of applications in nanomedicine such as drug delivery, biosensing, diagnostics, and theranostics.

#### 2.1.1. Bio-Imaging

Due to the extremely heterogeneous nature of cancer, antineoplastic therapies would take great benefit from precise and personalized approaches. The first step to plan a targeted strategy is the better understanding of the target itself from different points of view. Therefore, the accurate visualization of the tumor spatial hindrance, boundaries, and sometimes of the events occurring at the molecular level are the main objectives of nanoparticle-based diagnostics. The high electronic density combined with the SPR phenomenon allows the use of GNPs in assorted bioimaging methods capable of analyzing the tumor milieu from the nanoscopic to the macroscopic perspective.

Dark field microscopy (DFM), also called optical imaging, exploits the ability of some contrast agents to cause intense scattering of incident light [34]. GNPs’ high scattering coefficients can enhance the signal providing ultra-sensitive non-bleaching imaging compared to the conventional fluorescent dyes [69]. DFM of GNPs has been widely used for tumor cellular visualization aiming at different purposes, although the background scattering of biological tissues does not allow efficient in vivo imaging of deeper layers, mostly addressing its use in in vitro/ex vivo studies [70,71].

GNPs’ interaction with fluorescent molecules can be exploited in manifold ways to build bioimaging sensors based on FRET (Förster Resonance Energy Transfer). The application of FRET in biosensing is founded on the ability of GNPs to act as energy acceptors of electronically excited fluorescent donors [72]. It has been proven that this energy exchange among the two light-responsive moieties is strongly dependent on their mutual distance. Specifically, it is responsible for a fluorophore emission quenching for very short gaps (about 2 nm) or, on the contrary, can lead to a fluorescence intensity enhancement (Plasmon Enhanced Fluorescence—PEF) at intermediate distances (about 5 nm) [73]. Many authors exploited these properties to develop smart bioimaging sensors. Xu et al. designed a nanometric probe functionalizing gold bipyramids with Cy5.5-labeled hairpin-DNA sequences that recognizes telomerase. In this study, the number of DNA bases was carefully chosen to achieve a fluorescence “switch” that can distinguish between cancer (HeLa) and normal cells (LO-2) depending on the telomerase activity. The proposed gold nanosensors were able to effectively quench Cy5.5 fluorescence via FRET in absence of the substrate, exhibiting, on the other hand, a PEF effect after telomerase interaction, as the molecular dye reached the optimal distance from the gold surface [74]. Plasmon-enhanced fluorescence was further studied by Liu et al. constructing trypsin/folic acid decorated gold nanoclusters mixed with GNSs that selectively detect heparin in tumor-bearing mice, using FRET/PEF gold properties to visualize tumor masses directly in vivo [75].

The strong electromagnetic field surrounding the gold nanoparticles allows amplifying the Raman diffusion of molecules conjugated or adsorbed on the noble metal surface, resulting in increased sensitivity of SERS (Surface Enhanced Raman Scattering) imaging. This scattering technique, generally used for the study of bio-nano interactions [76], was also employed in imaging of gold nanoprobes on living animals by Qian et al. In their work, the accumulation of non-targeted and ScFv conjugated GNPs into Tu686 xenografts was investigated through in vivo SERS revealing in real-time the efficacy of the active targeting [77].

In the two-photon luminescence imaging (TPL) the contrast agent is stimulated simultaneously by two photons emitted by a high-power laser, focused by the objective of a multiphoton microscope. The energy absorbed as a result of stimulation is then emitted as fluorescence [78]. GNPs, and especially anisotropic gold colloids such as GNRs and GNSts, can be used efficiently in two-photon luminescence because the signal generated by them is significantly more intense and defined than that recorded with the classic fluorescent molecules [79,80].

Another widely used gold-based imaging method is photoacoustic imaging (PA), a high-resolution hybrid technique that allows the visualization of cells and tissues located in “deep” layers combining laser irradiation and ultrasound detection [81]. Due to SPR, gold nanoparticles lead to far greater optical absorption than conventional organic dyes, and for this reason, they are excellent contrast agents for photoacoustic imaging [82]. An ex vivo study of Mallidi et al. used GNSs PA to selectively detect the A431 squamous carcinoma cell line embedded in a gelatin tumor-model implanted in mouse tissue [83]. Moreover, PA could be employed to detect gold nanoparticles in vivo, as demonstrated by Song et al., which proved that gold nanocages can be suitably used for the PA-mapping of sentinel lymph nodes prior to biopsy [84].

Gold-based bioimaging can furthermore be performed through photothermal imaging (PTI). GNPs usage in PTI is a direct consequence of their photothermal effect exhibited after irradiation at wavelength matching (or close to) the plasmonic band [85]. As a result, anisotropic GNPs that possess a NIR-infrared plasmon are the most preferred shapes among the class to obtain safe and deep photothermal imaging [86].

Although the youngest gold-based imaging techniques are very promising and fascinating due to their high resolution and minimally invasive character, computed tomography (CT) remains among the most reliable imaging methods in tumor detection. Since it is based on the different X-ray attenuation between soft and dense tissues, the administration of a contrast agent that can enhance the visualization of the desired district is often necessary. Their high electron density, along with the possibility to achieve passive and/or active tumor targeting, makes GNPs a quite useful boon in contrast-CT [87]. In virtue of their in vivo applicability, gold-based PA, PTI and CT are maybe the most used techniques in image-guided phototherapy, a valuable tool in cancer theranostics.

#### 2.1.2. Image-Guided Phototherapy (IG-PT)

Gold nanoparticles might be powerful allies in tumor precision medicine, a field whose efforts in recent years have been focused on achieving patient-sewn solutions to tailor real-time therapeutic plans. Although the use of GNPs as contrast agents is in itself an advantageous opportunity to detect tumors in a minimally invasive manner, the possibility of availing us of gold-driven bioimaging to guide and gain a simultaneous therapeutic effect appears considerably more appealing. Although numerous examples of theranostic gold have already been reported in the literature, herein will be provided a slice of recent research focused on in vivo applications of GNPs image-guided phototherapy (IG-PT).

Jang et al. proposed multitherapeutic FRET nanoplatforms for the photothermal/photodynamic (PT/PD) treatment of SCC7 tumor xenografts. PEG thiol/peptide-coated GNRs were used to electrostatically load the fluorescent and photoactive molecule AlPcS4 so as to achieve gold-driven PT or PDT after 810 nm or 670 nm laser irradiation, respectively (Table 1). The detection of the nanosystems in the tumor site was thus carried out either with fluorescence (670 nm illumination) or infrared camera imaging (810 nm illumination), exploiting the “dequenching” of the fluorescent dye after the laser-triggered in situ release from the gold surface, or the heat generation due to the photothermal effect [88].

The value of IG-PT is not only limited to the real-time monitoring of the treatment, as it also offers the possibility of using predictive models that allow regulating treatment regimens according to the patient conditions in order to model the therapy in a personalized manner. This concept has been interestingly demonstrated by Von Maltzahn et al. who proposed the combination of GNRs CT imaging and computational sciences to predict the heat distribution in cancer (Table 1). In their study, GNRs were simultaneously employed as photothermal and contrast agents, using CT imaging to follow their accumulation in MDA-MB435 xenografts and to select the optimal irradiation parameters of irradiation. The nano-heaters’ PT, visualized during treatment through thermal imaging, was thus able to induce the complete tumor eradication, recording superior survival percentages [89].

Although IG-PT can lead to efficient in vivo tumor ablation, in view to obtain more energetic treatments with reduced risks of cancer recurrence, gold PT can be reinforced by supplementary antineoplastic strategies. A fascinating example can be found in a very recent work of Dai et al. Here, chitosan coated GNRs with highly controlled architectures were employed to synergistically treat breast cancer xenografts by combining PT and gene therapy. The biodistribution of the nanosystems, accomplished by PA imaging, was used as guidance to select the photothermal settings, achieving photothermal imaging-guided PT. Data furthermore showed that the efficacy of gold PT in slowing down the tumor progression was enhanced by gene therapy, reaching a considerable reduction of tumor volumes [90].

The use of GNPs along with other contrast agents was also explored to obtain a multi-modal IG-PT. By hybridizing gold-based nano constructs, researchers managed to produce platforms that can rely on different kinds of imaging approaches for the detection and the personalized therapy of solid tumors. In recent times, Li et al. proved that by encapsulating indocyanine green into the silica coating of gold nano bipyramids, dual-mode imaging (fluorescence/PA) of cancer can be realized. In this study, fluorescence acquisitions were merged with photoacoustic outputs to establish the most convenient PT time point as a function of the biodistribution data observed (Table 1). Photothermal imaging-assisted PT was hence performed, providing very encouraging results on A375 malignant melanoma xenografts regression [91]. Furthermore, a triple imaging theranostic system was formulated by Parchur et al., who designed an optical/MRI/CT nanotool composed of rare-earth and gadolinium doped GNRs for the PT of orthotopic CC531 implants. This time, CT imaging allowed monitoring the administration through a portal vein catheter as well as to guide the PT, supervising the thermal ablation procedure in real-time [92].

The examples reported are only a few among the many promising results realized in gold IG-PT. The possibility to combine the optoelectronic properties of GNPs with the advantages of passive and active targeting might lead to further progress in this field. Moreover, the hybridization of GNPs with other contrast agents, as well as the chance to use gold nanoparticles as reliable drug delivery systems, might pave the way to multi-modal theranostic tools for the image-guided multi-therapy of cancer.

### 2.2. Superparamagnetic Iron Oxide Nanoparticles (SPIONs)

Superparamagnetic iron oxide nanoparticles (SPIONs) are FDA approved materials consisting of Fe_3_O_4_ (II,III) oxide nanocrystals that have aroused great interest and exceptional achievements in targeted drug therapy [13,93,94,95,96] magnetic hyperthermia [97,98], focused ultrasound [99,100] and contrast agents for magnetic resonance imaging [35,101] (Figure 2). SPIONs are usually smaller than 30 nm in diameter and their magnetization and relaxation rate can be tuned according to the desired application. In particular, they can alter the T_1_ and T_2_ relaxation rates of fluids in the surrounding volume and the altered relaxation rate can be utilized for contrast enhancement in T_1_ and T_2_ weighted magnetic resonance imaging (MRI) for detection and imaging of tissues [102].

Various protocols have been established to produce SPIONs for biomedical applications, ranging from mechanical grinding and biomineralization to sol-gel synthesis, coprecipitation and ultrasound-assisted bottom-up approaches. Even if, for the most common SPIONs employed in MRI, size distribution is about 20 nm, the huge specific surface of these nanoparticles thermodynamically induces aggregation into larger particle clusters, thus implying that surface stabilization is mostly required. Additionally, Fe-OH surface groups at the SPIONs surface are reactive groups that afford to oxidized heterogeneous clusters in air or acidic aqueous dispersion [103]. As a consequence, surface stabilization of these nanomaterials is a crucial step to obtain nanomaterials suitable for biomedical applications.

Apart from the magneto-responsive core, particle size and coating can also be designed for a specific field of application. As magnetization and size impinge on the passive physical accumulation of SPIONs into the tumor mass, including magnetic targeting, surface functionalization significantly affects their stability in aqueous environments under physiological conditions and might provide SPIONs with smart properties in terms of sensitivity toward selected chemical or physical stimuli and active recognition of target cells, tissues or organs. Conventional surface functionalization of SPIONs consists of post-functionalization procedures by covalent or physical conjugation of polymer chains that leads to polymer-coated SPIONs of undefined structure. This kind of approach leads to self-assembled core-shell nanoparticles whose hydrophilic colloidal shell has the function of preventing oxidation and aggregation in physiological media. SPIONs for medical applications are typically coated using amphiphilic copolymers, whose hydrophobic domains capped SPIONs’ surface, giving rise to nanosystems with superior structural and magnetic features compared with their hydrophilic end-capped parent counterparts. Using this approach, SPIONs are completely covered by a collapsed hydrophobic film followed by covalent-bonded hydrophilic polymer chains that represent the particle–environment interface, thus influencing all interfacial phenomena occurring after the administration in humans. Usually, amphiphilic copolymers bearing lone-pair electron donors such as carboxylates, phosphates, thiols and hydroxyls may bind the surface of SPIONs, since magnetite has available LUMO which exhibit Lewis acid behavior [104]. Hence, copolymers carrying these functional groups can strongly stabilize the outer shell and have been extensively used to synthetize SPIONs with improved stability and biocompatibility [104].

Among these, poly(2-hydroxyethyl aspartamide) (PHEA) derivatives, where discrete poly(ethylene glycol) (PEG) and aliphatic side chains have been employed as functional side chains, have been successfully explored to stabilize the SPIONs’ interface and to improve long term circulation in vivo [105]. Using self-assembled amphiphilic copolymers, PEGylation results as a valid strategy to obtain thermodynamically stable colloids in PBS pH 7.4 for a long time (up to five days). Besides, they exhibited high biocompatibility due to the presence of PHEA backbone, a polymer with huge potential as a plasma expander. In a similar way, a branched amphiphilic 1,2-polybutadiene-PEG conjugate was used to obtain stable PEGylated SPIONs using a redox process [106]. PEGylated squalene-grafted-inulin amphiphiles capable of self-organizing into nanocarriers once placed in aqueous media have also been used, giving rise to superparamagnetic architectures endowed with stealth-like behavior and excellent physicochemical stability [14]. In particular, inulin, a low molecular weight natural polysaccharide (Mn < 3000 Da), was firstly modified in the side chain with primary amine groups, followed in turn by conjugation with squalenoyl derivatives and PEGylation by imine linkage. Polymer-coated SPIONs were synthesized by its spontaneous self-assembling onto a magnetite surface involving hydrophobic–hydrophobic interactions between the metallic core and the squalenoyl moieties. In addition, here the polymeric shell was exploited to load a high amount of doxorubicin, thus circumventing the very low loading capability that characterizes naked SPIONs. They displayed enhanced cytotoxicity and uptake abilities towards HCT116 cells under the influence of external magnetic fields, implying that surface coating would allow effective magnetic targeting and suitable therapeutic potential combined with MRI contrast properties.

Using another approach, SPIONs can be directly coupled with drug-loaded polymers of macromolecular prodrugs in order to obtain covalent-functionalized magnetic nanoparticles for combinatorial monitoring and chemotherapy of solid tumors [107]. For instance, Fu et al. presented hyaluronic acid and doxorubicin-based macromolecular prodrugs prepared by an acid-labile hydrazone linkage, and then conjugated with amine-modified iron oxide nanoparticles by a common carbodiimide mediated coupling reaction [108]. These SPIONs-polymer conjugates have the characteristics of good water dispersibility, superparamagnetic property and high magnetic relaxivity for magnetic resonance imaging, together with enhanced cellular uptake and specific accumulation in human hepatocellular liver carcinoma HepG2 cells due to specific biological recognition of hyaluronic acid. Besides, environment-sensitive SPIONs (i.e., pH- and glutathione-sensitive coatings), able to selectively release drugs inside cancer cells, can be synthetized by carefully designing the polymeric coating. For instance, polymers containing thiols [109] or protonable functional groups [109], at least in principle, provide a sufficient driving force to induce conformational changes during intracellular trafficking in the tumor microenvironment (passing from neutral to acidic pH or from normal to reducing conditions) so as to provoke massive drug release in situ, thus limiting multidrug resistance and off target side effects. pH-sensitive poly(β-thioproprionate)-coated SPIONs have been decorated with folic acid, used as targeting agents active against breast cancer, and doxorubicin was also loaded on the polymeric shell [109]. They suppressed tumors more effectively in the presence of static magnetic field than in the absence and proved capable of producing good MRI data in vivo. Redox sensitive, folate-conjugated multiblock polymer-coated γFe_2_O_3_ nanoparticles, containing a glutathione (GSH) sensitive PLGA-PEG-PLGA-urethane-SS- block copolymer coating, were used to deliver a high amount of doxorubicin in cancer cells and in a glutathione-dependent fashion (about five times higher in the presence of 10 mM GSH) [108]. This combinatorial approach can help in achieving a better therapeutic effect with minimal side effects of chemotherapy.

Mostly, surface functionalization of SPIONs for cancer theranostic applications follows complex protocols that yield to nanotools whose characteristics are neither consistent nor suitable for industrial production. Hence, the development of much more controlled and industrially scalable surface functionalization protocols is needed. Innovations involving shape and targeting technologies, combined with new strategies to focus static magnetic fields in selected human body districts, have the potential for improving the efficacy of theranostic applications of systemically administrated SPIONs.

#### 2.2.1. Magnetic Targeting by Superparamagnetism

Superparamagnetism is the property of small ferromagnetic or ferrimagnetic particles of reversibly switching their average magnetization under the influence of an external magnetic field [103]. Typically, superparamagnetic nanoparticles have an anisotropic arrangement of their magnetic moments and tend to align them when exposed to a magnetic field (magnetization) in the direction of the field. Besides, when the field is removed, they also return to zero (hysteresis). As a rule, magnetic hysteresis is a non-equilibrium process, implying that macroscopic magnetization tends to vanish with a characteristic relaxation time after the removal of the external magnetic field. SPIONs exhibit a collective magnetic domain, and as a consequence, an applied magnetic field has the same effect on the whole magnetite particles, and their mobility can be manipulated from outside the body for in vivo medical applications.

Magnetic targeting was proposed for the first time by Freeman, who envisaged magnetic nanocarriers to target tumor tissues and selectively release their payload directly in the site of action, thus avoiding unspecific delivery of toxic drugs into healthy organs [107]. The idea is to load biocompatible carriers who reversibly respond to external magnetic stimuli with anticancer drugs and, after systemic administration and proper accumulation to the tumor mass due to the application of a magnetic gradient, release them only at the target site (Figure 2). However, once the drug-loaded carrier is localized at the site of action, the drug should be released by peculiar stimuli of the tumor microenvironment such as enzymatic cleavage, pH, temperature or osmolarity changes. Magnetic targeting by superparamagnetic carriers not only significantly circumvents undesired side effects by addressing biodistribution to the target site, but also allows a dosage reduction while keeping the local concentration within the therapeutic window [103,110].

Many research groups have pursued their studies in comparative analysis of anticancer drugs bioavailability at the tumor site after either enhanced permeability retention (EPR) effect-based administration (passive targeting) or magnetic targeting-based administration. Besides, many works have focused their attention on differences between magnetic targeting and receptor-mediate targeting (active targeting) as well as the synergistic enhanced accumulation of nanosystems owing to combinations of them. Even if there are contradictory data around the synergistic effect between active and magnetic targeting, several works have concluded that magnetic targeting allows reaching higher drug accumulation in vivo after systemic administration in mice. For example, RGD-containing paclitaxel-loaded SPIONs were proved capable of accumulating in colorectal carcinoma (CT26 cell line) more efficiently under the effect of an external magnetic field placed outside the tumor and also by comparison with PLGA-based nanoparticles [111]. Results pointed out that the combinatory approach (i.e., magnetic targeting and RGD-mediated targeting) had the most promising outcome, suggesting a pivotal rule of these two synergistic targeting strategies. In particular, apart from the most prolonged survival rate shown, SPIONs accumulation in the tumor was roughly eight times higher than that observed for the passive targeting due to PLGA-based nanoparticles administration. Huang et al. proposed hybrid SPIONs for gastric cancer theranostics with a similar combinatorial targeting approach and comparable results (Table 1) [112].

#### 2.2.2. Magnetic Resonance Imaging (MRI)

The importance of SPIONs for cancer theranostics focuses on three main properties: magnetic targeting and selective drug delivery, magnetic hyperthermia and, the last but not the least, contrast agent for MRI (Figure 2). MRI is one of the most powerful non-invasive imaging techniques employed in the clinic to image and monitor the progression of pathologies such as cancer [113]. It is based on the relaxation of protons in tissues that are strongly influenced by the characteristics of the chemical surroundings. Nanosystems that enhance proton relaxation of specific tissues compared to that in the surrounding tissues may serve as contrast agents in MRI applications [114].

In principle, SPIONs have a collective magnetic moment which tends to align with the axes of magnetization induced by an external magnetic field, and magnetic relaxation phenomena owing to the removal of the magnetic field depend on both Néel and Brownian relaxation [115]. Usually, SPIONs are dispersed in a complex aqueous environment in tissues and the return of the magnetization to the equilibrium is characterized by a typical relaxation times (τ) which is influenced by many factors such as viscosity, temperature, hydrodynamic size, and magnetic core size. However, the MRI capability strictly depends on the distortion of the longitudinal (*T*_1_) and transverse (*T*_2_) relaxation of the surrounding nuclei rather than the inherent relaxation properties of SPIONs. This is because the magnetization of SPIONs in the direction of the external magnetic field enhances the magnetic flux, thus eliciting rephrasing of the surrounding protons under the effect of a greater local magnetic moment. Under these conditions, the proton magnetic moments of water spin in an external magnetic field and absorb energy at a frequency which is proportional to the external magnetic field and the nuclear gyromagnetic ratio. The efficiency of a MRI contrast agent in reducing *T*_1_ and *T*_2_ is measured by its relaxivity (*r*), defined as the slope of the relaxation rate as a function of the equivalent ion concentration in mM [115]. SPIONs are usually prone to increase the *r*_2_ values, thus acting as a valid contrast agent in *T*_2_ weighted MRI applications.

High *r*_2_ values can be obtained by decreasing the water diffusion coefficient of polymer-functionalized SPIONs or increasing the hydrodynamic diameter of the magnetic core of self-assembled amphiphilic polymer-SPIONs nanosystems. As found by Parker and co-worker, the *r*_2_ values of water’s protons surrounding poly(acrylic acid)-g-poly(styrene)-coated SPIONs increases with increasing SPIONs diameter reaching 555 mM^−1^ S^−1^. Even if extremely high *r*_2_ values can be found for complex nanomaterials consisting of agglomerated SPIONs dispersed in a polymer matrix such as polymerosomes (*T*_2_ relaxivity > 600 mM^−1^ S^−1^) [36,116,117], these nano-sized architectures are quite difficult to manipulate to develop pharmaceutical grade products. In fact, a high control of the composition and physicochemical characteristics are warmly desired in view of clinical applications.

Hydrophobic SPIONs synthesized from thermal decomposition of iron salts must be rendered hydrophilic for their application as MRI contrast agents. This process requires surface-engineering protocols employing hydrophilic coatings such as polymers or small molecules. Two parameters can impinge on the magnetic behavior and relaxivity of the resulting hydrophilic SPIONs: the functional group of the surface moiety and the protocol for the surface engineering. Clinically approved SPIONs are simple large nanoparticles coated with polymers such as polysaccharides or synthetic polyacrylates [118]. However, they have been withdrawn from the market after the introduction of gadolinium-based contrast agents, since the latter provide a positive contrast remarking evidence of unsatisfactory efficacy of SPIONs in diagnosis and cancer monitoring.

Huang and co-workers have developed a one-step facile surface chemistry approach for the surface functionalization of SPIOs with a lung cancer-targeting peptide [119]. The hydrophobic surfactants on the as-synthesized SPIOs were displaced by the peptide containing a poly(ethylene glycol)-tethered cysteine residue through ligand exchange. The resulting SPIOs were biocompatible and demonstrate high *T*_2_ relaxivity. The nanoprobes were specific in targeting α_v_β_6_–expressing lung cancer cells, as demonstrated by MRI experiments. In a similar approach, Wang et al. synthesized bovine serum albumin (BSA) surface functionalized hydrophobic SPIONs via ligand exchange [120]. The longitudinal and transverse proton relaxation rate values of the BSA-SPIONs were determined to be 11.6 and 154.2 mM^−1^ S^−1^, respectively. The *r*_2_/*r*_1_ ratio of 13.3 suggests a potential application of these nanosystems as T2-weighted MRI contrast agents in pancreatic cancer. Interestingly, in a systematic work by Smolensky et al., it was found that relaxivity of surface functionalized SPIONs highly depends on the kind of pendants and the protocols adopted during the functionalization step (Table 1) [121]. In particular, they found that anchoring catechol function-bearing PEG chains, such as dopamide and 2,3-dihydroxybenzamide, yield to a retention of relaxivity if compared with the native naked hydrophobic SPIONs. On the contrary, carboxylates and especially dopamine alone significantly decreased the relaxivity of the SPIONs. Besides, surface functionalization of SPIONs with PEG chains was much more conservative in terms of relaxivity if functionalization occurred by biphasic ligand exchange protocols instead of stripping procedures. This might be ascribed to changes in morphologies. In all these applications, the tailoring of the SPIONs surface is mandatory, not only to improve biocompatibility, solubility, and stability, but also to ensure a small particle size distribution (below 150 nm) after surface engineering and to preserve excellent magnetic properties.

#### 2.2.3. Direct Magnetic Imaging and Cancer Ablation

The improving of MRI sensitivity and the therapeutic function of SPIONs in cancer theranostics has received considerable attention, especially for the mini-invasive eradication of solid tumors by magnetic hyperthermia. For cancer therapy, hyperthermia refers to a rise in temperature up to 45 °C, which yields to cell death through the activation of a tandem of pro-apoptotic and apoptotic signaling cascades [122]. When the temperature is above 46 °C, irreversible thermoablation would occur by means of necrosis [123]. SPIONs are capable of transforming electromagnetic energy into heat, thus the heat generated under an external alternate magnetic field, attributable to their hysteresis dissipation [124], can used to induce selective ablation of a tumor mass. Hyperthermia at lower temperatures can be also used as a sufficient insult to increase susceptibility of cancer cells to other treatments such as surgery, radiotherapy, chemotherapy, and hormonal therapy [125]. In 2010, a SPIONs formulation (NanoTherm^®^) was approved for the treatment and imaging of glioblastoma through MRI-guided hyperthermia. NanoTherm^®^ consists of aminosilane-functionalized SPIONs of about 15 nm in diameter. It was approved for direct intratumoral injection, thus allowing imaging of the tumor mass by MRI and the treatment by applying an alternating magnetic field to reach temperatures of about 45 °C [126]. These surface-functionalized SPIONs are simple, and there are currently several pilot studies for the treatment of breast, prostate, pancreatic and esophagi cancer [127,128,129]. Besides, in 2018, the FDA approved NanoTherm^®^ for tumor ablation of prostate cancer. Recently, PEGylated SPIONs nanocubes were proved capable of impeding tumor growth through magnetic hyperthermia when intratumorally injected into epidermoid carcinoma xenograft implanted mice [130]. Herein, it is reported that off-target temperature levels retained normal values, whereas the tumor tissue reached high temperatures. Results also showed that these nanocubes act much better when located in the interstitial extracellular space instead of at the intracellular level, since they exhibited lower heating capabilities upon cell uptake. Magnetic hyperthermia has also been used to trigger both hyperthermia and locoregional drug release. Poly(vinyl alcohol)-coated SPIONs have been successfully developed to co-deliver doxorubicin and paclitaxel in a magnetic-sensitive way. When these SPIONs were subjected to an external 50 kHz magnetic field, the release rate of both drugs increased. This shows great potential for SPIONs use as on-demand drug release vehicles in combination with hyperthermia [131].

In principle, other techniques might be used for MRI-guided thermal ablation by SPIONs such as radiofrequency (RF) ablation, laser interstitial thermotherapy (LITT), microwave (MW) ablation, high-intensity focused ultrasound (HIFU), photodynamic therapy (PDT), phototherapy (PTT), and cryoablation [132] (Figure 2). Among these, PTT exploits a visible or NIR laser light source to activate thermal heating of SPIONs through photon absorption. Typically, SPIONs have been functionalized with functional nanomaterials in order to yield multilayer nano-hybrids with a peculiar combination of theranostic properties [133]. Gold-coated SPIONs or graphene oxide (GO) nanocomposites are employed in photothermal therapy due to their ability to absorb NIR wavelengths, a region of the electromagnetic spectrum that offers optimal tissue penetration. Smart nanotheranostics platform was constructed using engineered GO and the in situ growth of ultrasmall SPIONs [134]. The construction of such novel tools show great potential in tumor theranostics, especially for T_1_-weighted MRI-guided and pH-sensitive chemo-phototherapy. A controlled surface functionalization of SPIONs with gold nanoparticles was attained thus leading to complex hybrids consisting of a superparamagnetic core and a NIR-responsive gold shell. Using this approach, Abed et al. synthesized nanotheranostic agents for magnetically targeted cancer photothermal therapy (Table 1) [135]. The anticancer efficacy of this nano-hybrid system was established in vivo on colorectal cancer models using a xenograft of CT26 cells. The feasibility of magnetic targeting was studied, placing a magnet outside the tumor mass during biodistribution phenomena and measuring the accumulation of the nanosystem over time by MRI. They demonstrated that the nano-hybrid can accumulate in the tumor mass after 3 h and that a complete remission of tumor growth was obtained after irradiation with a suitable near infrared (NIR) source. In another approach, gold and iron oxide hybrid nanoparticles (HNPs) synthesized by thermal decomposition were bio-functionalized with a single chain antibody, scFv, that binds to the A33 antigen present on colorectal cancer cells [136]. Cellular uptake studies showed that A33-expressing cells take up the A33scFv-conjugated HNPs at a rate five times higher than cells that do not express the A33 antigen. Then, 808 nm laser irradiation implies that approximately 53% of the A33-expressing cells exposed to targeted HNPs are killed after a six-minute laser treatment at 5.1 W cm^−2^, while <5% of normal cells were killed under the same treatment. Again, highly selective cancer cell death was achieved by SPIONs/gold hybrids engineenered with epidermal growth factor receptor [137].

**Table 1:** Comparison of selected nanoplatforms for theranostc applications.

Nanoplatform	Functionalization	Theranostic Approach	Use	Outcome	Ref.
GNRs of 34 nm and 3.7 aspect ratio	SH coupling of MeO-PEG-SH and surface coating of photoactives	IG-PT (FRET-PT/PD)	Squamous cell carcinoma	95% tumor reduction under the guide of NIR imaging	[88]
GNRs of 40 nm and 3.5 aspect ratio	SH coupling of MeO-PEG-SH	IG-PT (CT-PT)	Breast cancer	~2-fold higher CT contrast, blood half-lives of ~17 h, ~7% ID/g tumor accumulation and total ablation of tumor mass	[89]
Silica-GNPs hybrids of 18 nm	NH_2_ surface functionalization and amide coupling with ICG	IG-PT (PA/FL-PT)	Skin cancer	Blood half-lives of ~24 h, accumulation in different organs and total remission of tumor mass under the guide of PA/FL imaging	[91]
SPIONs of 11 nm	Suface coating with PEI, PEG and folic acid	Magnetic targeting (MRI-DDS)	Breast cancer	MRI contrast, ~6.9-fold higher drug accumulation in tumors and total remission of tumor mass	[112]
SPIONs (γ-Fe_2_O_3_ and Fe_3_O_4_) of 9 nm	PEG surface coating	MRI	Solid tumors	MRI contrast ~4-fold higher than commercial counterpart	[121]
SPIONs-GNPs hybrids of 23 nm	Au surface deposition and folic acid coupling	IG-PT (MRI/CT-PT)	Colorectal cancer	Total remission of tumor mass under the guide of MRI, high spatial resolution MRI of the tumor	[135]
C_3_N_4_ CDs of 5 nm with red emission	Surface amide coupling with NH_2_-PEG-Biotin	IG-PT (FL/Chemio-PT)	Breast cancer	Selective accumulation in tumor cells and 98% tumor reduction under the guide of FL	[21]
CDs of 5 nm	Surface amide coupling with NH_2_-PEG-Folate	IG-PT (FL/PD-PT)	Cervical cancer	Accumulation in tumors and reduction of mouse mortality under the guide of FL	[138]
Cu-doped CDs of 3 nm	Non-passivated	IG-PT (FL/PD-PT)	Melanoma	Blood half-lives of ~14 d, ~8% dose accumulation in tumor, total remission of tumors under the guidance of FL imaging	[139]
GO of 470 nm and 1 nm in thickness	Surface amide coupling of NH_2_-PEG-OMe	IG-PT (FL/PD-PT)	Metastatic breast cancer	Accumulation in tumor, spleen and liver, 75% tumor inhibition under NIR exposure	[140]
GO-SPIONs hybrids of 174 nm	Surface functionalization with SPIONs-NH_2_ via amidic coupling	IG-PT (MRI/PT)	Solid tumors	Improved *T*_2_ weighted MRI contrast and high NIR photothermal potential	[141]
GO-SPIONs hybrids of 50 nm	In situ surface seeding of SPIONs and PEG coupling	IG-PT (PA/MRI-PT)	Metastatic breast cancer	5% dose accumulation in tumor after 48 h, combined PA and MRI contrast, total remission of tumor mass under the guide of PA and MRI	[142]

### 2.3. Carbon Nanodots (CDs)

Carbon nanodots (CDs), discovered in the early 21st century, are 0D carbonaceous nanomaterials smaller than 10 nm, typically composed by carbon, oxygen, and hydrogen. CDs emerge as a promising nanoplatform for different applications, including drug delivery [143], bioimaging [144] and sensing [38]. In the field of theranostic nanomedicine, CDs have been demonstrated to have many appealing properties, such as tunable photoluminescence (PL), light-induced photothermal conversion, strong hydrophilicity and high biocompatibility (Figure 2) [145]. Multicolor fluorescence, from blue to near-infrared (NIR), has gained particular interest in the development of long-wavelength emissive CDs-based nanoplatforms as a new powerful strategy of fluorescence imaging (FLI) useful in cancer diagnosis. Carbon nanodots also proved to be excellent photothermal agents due to their capacity to convert NIR light in local temperature increase, applicable in cancer photothermal therapy (PTT) [146]. In light of these considerations, researchers focused their attention on identifying an effective synthetic strategy to achieve precise control in the performance of CDs, in terms of size distribution, surface and optical properties. As extensively reported in the literature, CDs could be produced by different routes classified in: top-down and bottom-up approaches (Figure 2) [39]. Top-down methods (e.g., laser ablation, electrochemical synthesis or chemical oxidation) consist in the production of CDs from pre-formed carbon structure, such as carbon nanotubes, fullerene or graphite. Although these approaches often lead to the formation of carbon dots with a well-defined structure (e.g., graphitic structure), they are poor fluorescent without successive surface passivation [147]. On the contrary, bottom-up methods, with an appropriate selection of the reaction conditions (e.g., reagents, solvent, pressure, temperature), are more versatile and effective to obtain high emissive carbon dots [148]. These approaches (e.g., hydro/solvothermal, microwave-assisted or ultrasonic synthesis) exploit the carbonization of organic molecules or biomass such as citric acid, amino acids or natural materials. However, bottom-up methods do not allow precise control of the size of the products, often leading to the formation of an extremely heterogeneous product. Only a few reports have investigated synthetic protocols to produce CDs via controlled solvothermal conditions (i.e., temperature and pressure), suggesting the possibility of tuning their physicochemical properties by pressure and temperature-dependent procedures [20,149]. As a consequence, post-synthetic purification, through dialysis or size exclusion chromatography (SEC), is always necessary to obtain highly homogeneous carbon dots. The bottom-up techniques offer many advantages against top-down approaches, also arousing the attention on many studies for the possibility of easily introducing external atoms (e.g., nitrogen, sulphur and boron) as doping elements of the CDs core. Besides, many organic waste or byproducts can be employed in bottom up synthesis of CDs in order to produce highly fluorescent CDs by eco-friendly routes [150]. In particular, the doping of CDs core with nitrogen has proven to significantly change the optical properties, enhancing PL, red shift and light absorption capacity (Figure 2). The synthetic approach and dopant-elements highly affect the determination of core and surface characteristics of CDs as well as optical and morphological properties.

On the basis of the core structure, CDs are classified as: graphitic, amorphous and doped carbon dots. Graphitic CDs, generally produced by top-down synthetic methods, are quasi-spherical graphite-like structures composed of crystalline layers of carbon stacked by a few nanometers, in contrast with graphene quantum dots, mainly based on nano-sized fragments of graphene monolayers. The other type of carbon dots comprises an amorphous spherical structure based on a core of sp^2^/sp^3^-hybridized carbons [151,152]. C_3_N_4_ carbon dots are a peculiar class of nitrogen-rich CDs obtained at a high level of N-doping, which have shown excellent optical properties. They are characterized by the repetition of carbon nitride structures and can be organized in a graphitic structure (g-C_3_N_4_) or in a crystalline core (β-C_3_N_4_) as a function of the percentage of nitrogenous precursors [148]. Another predominant intrinsic factor of CDs is represented by their surface functional groups that can be very variable (e.g., carboxyl, hydroxyl, amino and amide groups), making CDs highly versatile and easy to functionalize. The surface state, extremely influenced by the adopted synthetic scheme, affects more important aspects of CDs’ profile, such as FL, directional capacity and biocompatibility [20,23,153,154,155,156]. The concept of surface functionalization as a new strategy to improve optical properties of CDs was proposed for the first time by Sun et al. in 2007. In this work, the passivation of CDs surface with amino PEG1500 has been demonstrated as a valid approach to favor the red shift of the emission peak as well as to increment the QY. Another important aspect is the marked biocompatibility of CDs, a strength point in the use of the latter for the engineering of theranostic nanosystems. Besides, the ultra-small size of CDs, often less than the cut-off of renal excretion (5 nm), ensure bioelimination of most CDs after administration, thus making them biodegradable and suitable for biomedical applications, avoiding the problem of unspecific organ accumulation and long retention time in reticuloendothelial systems [157]. However, their rapid bioelimination in vivo could also jeopardize their pharmacokinetic profile favoring their rapid renal excretion and lowering bioavailability of their drug payload. As a consequence, surface passivation of CDs could play an additional role for their possible application in vivo, increasing the average size, and in some cases, improving their biocompatibility and reducing RES clearance [158]. Thus, the CDs with intrinsic theranostic properties and suitable engineering have shown considerable potential for application in cancer diagnosis and therapy [159]. For instance, surface-engineered N-doped CDs passivated with bifunctional amino-PEG_2000_-biotin pendants via a controlled click chemistry approach have demonstrated a valid strategy to improve their biocompatibility and, increasing the average size to about 8 nm (>renal cut-off), to potentially ensure a better biodistribution in vivo [21].

Additionally, C_3_N_4_ CDs synthesized by bottom-up route show high emissive and photothermal conversion capacity, which were improved after functionalization, confirming the efficacy of a strategic synthesis and surface passivation of CDs. Moreover, the functionalization of CDs with biotin as a pedant confer targeting property towards MCF-7 and MDA-MB-231, which favor their accumulation in cancer cells, improving their therapeutic efficacy. Thus, suitably engineered CDs with intrinsic theranostic properties have shown considerable potential for application in cancer diagnosis and therapy.

#### 2.3.1. Optical Properties

Due to their own tunable fluorescence, high quantum yield and exceptional good photostability, carbon nanodots have aroused particular interest in optoelectronic, green energy and imaging applications [148,156,160]. They are relevant in many biomedical fields since photoluminescent carbonaceous CDs exhibit low toxicity, especially if compared with other quantum dots, and their emission properties can be tuned within the biologically transparent window (600–1100 nm) [161,162]. This provides an exceptional contrast in fluorescence imaging for in vivo applications such as tumor diagnosis and imaging. However, it is necessary to make clear the photoluminescence/structure relationship in CDs to improve their quantum yield (QY) and modulate it as a function of the application. It is known that the penetration ability of different light sources across living tissues is different and, in particular, that only long wavelength emissions (from red to NIR) can reach deep tissues provoking negligible damages [157]. Hence, tuning the CDs emission to the NIR region is desired and should be required for cancer theranostics applications.

Currently, the development of NIR-emitting CDs is mostly fortuitous since a rational design of their photoluminescence on paper is precluded. Although some correlations between surface state or size and emission spectra of CDs have been reported, their mode of emission may not be adjusted by simply combining established starting monomers or bulk materials. Up to now, several PL mechanisms have mainly been suggested to originate from surface state, conjugated structures and the formation of special structure sites on the surface [163]. However, only two main radiative mechanisms have been reported to allow tuning CDs photoluminescence: (i) quantum confinement (related to the size of the carbon core) and (ii) surface state (related to the oxidation degree of the surface). When quantum confinement prevails on other mechanisms, the emission band of CDs will undergo a red-shift by increasing the carbon core size [164].

On the contrary, a red-shifted emission band can be obtained, increasing the amount of carboxylic functions (oxidized species) at the CDs surface [156,164]. This viewpoint is that the surface state of CDs impinges on the radiative recombination of the CDs surface-confined electrons and holes that are responsible for the photoluminescence phenomenon in CDs. Indeed, surface functionalization of CDs with small molecules and polymers was found to induce an additional dimension in controlling CDs fluorescence together with their properties at the nanomaterial/cell interface. For example, Lin and co-workers have demonstrated that surface functionalization of CDs with imidazole can enhance chemiluminescence of nitrogen-doped CDs [165]. Besides, the introduction of heteroatoms as doping agents can lead to red-shift in the emission band of CDs because of the narrower energy gap at the surface [166,167]. Jiang et al. synthesized multi-color N-doped CDs from three isomers of phenylenediamine in solvothermal condition (ethanol, 180 °C, 12 h), proposing a possible correlation between emissive capacity and nitrogen content. The use of meta-, orto- or para-phenylendiamine conduce to the formation of carbon dots emitting in the blue, green, and red region, respectively. This difference in the emissive properties was accompanied with an increase of nitrogen content from 4% to 16%. Moreover, an augment of the average size from 6 nm to 10 nm in parallel with the red-shift of the emission band was also observed [168]. Messina et al. synthesized and compared low N-doped CDs and high N-doped CDs, assessing how the level of N-doping effected their core structural and emissions properties. Low N-doped CDs have shown an emission band in the blue range of spectrum and displayed a graphite core, while high N-doped CDs exhibited a β-C_3_N_4_ crystalline core and fluorescence of blue and green [169].

As widely reported in the literature, the choice of solvent reaction could also be exploited to modulate the fluorescence properties of carbon dots. Carbon dots synthesized in water often show blue emission, despite the synthesis of CDs in DMF, typically from urea and citric acid, in comparison with the use of polar solvents, improves the redshifted emission and absorption [170]. Another interesting work demonstrated the influent role of pressure reaction condition on the optical properties of CDs, through the modulation of size distribution and surface functional groups. In detail, the same scheme of reaction was replicated at 8, 13 and 18 bar, in order to evaluate the influence of pressure parameter on CDs profile. The increased pressure conducted to an increment of the average size and surface carboxyl groups, which resulted in a redshift of the emission; proving the key role of another reaction parameter in the determination of CDs features [20]. In order to modulate the optical properties to obtain performing CDs in cancer theranostic, functional groups on the surface of CDs are an important factor to take into consideration [171]. These fascinating emission properties have been widely exploited for the development of fluorescent markers and imaging agents for both in vitro and in vivo applications.

The combination of fluorescence imaging, photoacoustic imaging, and PTT have attracted increasing interest because they are noninvasive and provoke negligible tissue damages [172,173]. These techniques require nanomaterials that have strong fluorescence and high photothermal conversion within the biological transparency window [146]. The main absorption bands of CDs are typically in the green region of the spectrum, thus tuning these bands to the red-to-NIR region to obtain acceptable performances for PTT remains challenging [174,175]. For instance, Lan et al. reported CDs with a maximum absorption band at 526 nm and a photothermal conversion efficiency of 58.2% under a 635-nm laser at 2 W cm^−2^ power density [176]. Zheng et al. synthesized NIR-emitting CDs with maximum absorption at 370 nm and a photothermal conversion efficiency of 38.7% under an 808-nm laser at the same power density [177]. Nitrogen-rich CDs were found to undergo a significant red-shift in the absorption bands, thus improving photothermal conversion under NIR light source irradiation.

Qu’s group have also demonstrated that the co-doping of nitrogen-rich CDs with sulfur atoms by the solvothermal carbonization of citric acid and urea in dimethyl sulfoxide (used as sulfur source) yield to CDs with broad and strong absorption band in the red-to-NIR region with a maximum absorption coefficient at 600 nm and a mass absorption coefficient in the red to NIR region that is much higher than that of graphene oxide [146]. Besides, strong NIR emission at 720 nm and extremely high photothermal conversion efficiency (59.19%) were simultaneously achieved under 655 nm diode laser irradiation. They demonstrated that such CDs can accumulate in tumor tissues in vivo, thus provoking selective light triggered damages to the target site in combination with high photoacoustic performance. Overall, CDs are endowed with a peculiar combination of optical properties that make them suitable for transfer to clinical medical practice in view of personalized and precise approaches.

#### 2.3.2. Bioimaging and Biosensing by Carbon Nanodots

Due to their photoluminescence and photostability, especially in the biologically transparent window (600–1100 nm), CDs are considered the most promising nanosystems ensuring bioimaging and biosensing both in vitro and in vivo. Bioimaging and biosensing of tissues, cells and molecular species are key factors in the diagnosis and precise treatments of diseases such as tumors. The advantages of CDs as bioimaging agents are mainly attributed to the lack of metal ions, which might adversely affect their use in humans due to bioaccumulation phenomena. Indeed, they result in highly biocompatible nanomaterials with tunable absorption and emission spectra suitable for fluorescence imaging applications. CDs are efficiently and rapidly excreted from the body after injection in mice with a clearance rate ranked as: intravenous > intramuscular > subcutaneous. CDs usually have relatively low retention in the reticuloendothelial system (RES) and showed high tumor-to-background contrast, thus they are suitable for tumor imaging applications. Besides, thanks to their ability to respond to environment changes (e.g., pH, ion strength, metals, etc.), they can be used as sensors. While rare-earth-based nanoparticles suffers from heavy metal elements and relatively short lifetimes, CDs have manipulative luminescence lifetimes which can respond to specific stimuli, thus allowing fluorescence lifetime imaging (FLI) [178,179]. For example, the luminescence lifetimes of the CDs can be manipulated from nanosecond level to second level by introducing water in solution, thus promising potential applications of CDs in multi-lifetime channels biological imaging [178].

Various green-emitting biocompatible CDs have been successfully reported in the literature for theranostics studies [160,180]. For instance, Sahu et al. reported the synthesis of highly stable and photoluminescent CDs with a quantum yield (QY) of 26% by hydrothermal treatment of orange juice [181]. They showed that these CDs have low toxicity and can be used as excellent probes in cellular imaging. However, due to the high absorption abilities of bioelements within the blue-green region, the use of green-emitting CDs in clinics is precluded especially for cancer theranostics of organs and deep tissues. On the contrary, red-emitting CDs have huge potential in theranostics owing to the lack of interactions with living tissues and deep light penetration in vivo. Wang and co-workers have reported the green microwave-assisted synthesis of switch-on CDs-based fluorescent nanothermometry device for spatially resolved temperature measurements in living cells [182]. These CDs exhibit red fluorescence (λ_em_ = 615 nm) with high QY (15%). Then, an on–off fluorescent probe was prepared for detecting reduced glutathione based on aggregation-induced fluorescence quenching. Interestingly, the quenched fluorescence could be recovered by increasing temperature, and the CDs-GSH mixture could behave as an off–on fluorescent probe for temperature. Thus, red-emitting CDs can be utilized for “turn-on” fluorescent nanothermometry through the fluorescence quenching and recovery processes in living tissues. This is particularly interesting for cancer photothermal applications, since fine cell temperature measurements are required to control hyperthermia, and thus cell death.

Most of the reported CDs exhibit insufficient excitation and emission in red/near-infrared regions, which significantly limits their practical applications in biomedical assays and therapy. In recent years, extensive studies have been performed to produce CDs with intensified red/NIR excitation and emission by designed reactions and precise separations. In another interesting work, Liu et al. designed red/NIR emissive CDs with QY of 57% via an in situ solvent-free carbonization strategy [183]. One-photon and 2-photon cellular imaging was demonstrated by using the CDs as red/NIR fluorescence agent due to the high photoluminescence and low biotoxicity. A further study showed that the red/NIR CDs exhibit multiphoton excited upconversion fluorescence under excitation of 800–2000 nm, which involves three NIR windows (NIR-I, 650–950 nm; NIR-II, 1100–1350; NIR-III, 1600–1870 nm). Two-photon, 3-photon, and 4-photon excited fluorescence of the CDs under excitation of different wavelengths was achieved, which may push forward the application of the CDs in bioimaging. To improve the tumor targeting and uptake efficiency of CDs, Yang et al. developed an active tumor targeting imaging system by surface engineering of CDs with a tumor-homing penetration peptide named iRGD (CRGDKGPDC) [184]. CDs of about 3.5 nm in diameter were obtained hydrothermally using melanin as precursor and red shift emissive CDs were obtained after surface adsorption of iRGD macromolecules. Particularly, iRGD-CDs showed higher cellular uptake in vitro, while they presented higher penetration and accumulation in tumor tissue in vivo, leading to better tumor imaging efficacy. In 2019, Hao’s group synthesized second near-infrared emission CDs derived from watermelon juice via a hydrothermal route [157]. These CDs possessed emissions at 900–1200 nm with a QY of 0.4%, high biocompatibility, and rapid renal clearance, making them desirable contrast agents for fluorescence bioimaging and cancer theranostics application. These are an excellent example to prepare CDs with long-wavelength or multicolor emissions using biomass as carbon source, even if biomass-derived CDs are subject to relatively low QYs and few available precursors in comparison with CDs derived from organic compounds [185].

Poly(vinylpyrrolidone)-functionalized CDs with both excitation and emission in the NIR regions (λ_ex_ = 715 nm, λ_em_ = 760 nm) have been also explored by Rogach et al. as NIR fluorescence contrast agents in live mice [186]. They showed that a bright NIR fluorescence signal occurred in the stomach of the mouse under 671 nm laser excitation, implying that CDs can be easily distinguished from the background through 800 nm longpass optical filter in vivo. NIR emitting CDs could be clearly distinguished from the background with good contrast in vivo and, because of their NIR absorption properties, they are particularly advantageous for high-resolution in vivo imaging.

CDs can also be employed as contrast agents in photoacoustic imaging (Figure 2), that is a non-invasive optical and ultrasound imaging modality which offers depth tissues detection at high resolution. This opportunity is ascribed to the distinct NIR absorption, high extinction coefficient and non-radiative generation of local heat shown by CDs [186,187]. Wu et al. have designed bioeliminable CDs from microwave-assisted solvent-free decomposition of commercial honey and rapid surface passivation with organic macromolecules (e.g., polysorbate, polyethylene glycol) to give photoluminescent CDs of about 7 nm in diameter [188]. They displayed strong optical absorption in the NIR region, tiny size, rapid lymphatic transport and an exceptionally rapid signal enhancement (∼2 min) of the sentinel lymph nodes in mice. Hence, they proposed these CDs for faster resection of sentinel lymph nodes by means of photoacoustic imaging guidance in metastatic breast cancer. Qu’s group prepared NIR-CDs from solvothermal decomposition of citric acid and urea as carbon sources in dimethyl sulfoxide, and explored their potential as multimodal contrast agent using in vivo models [189]. CDs exhibited a broad and strong absorption band from red to NIR region with a maximum absorption coefficient at 600 nm and a NIR emission peak at 720 nm. The feasibility of using NIR-CDs as an efficient NIR light-triggered photoacoustic contrast agent was proved in vivo after intravenous injection into mice with 4T1 tumors. These CDs were uniformly accumulated in the tumor area through the enhanced permeability and retention (EPR) effect, exhibiting the maximum accumulation after 3 h, demonstrating that the NIR-CDs can simultaneously act as fluorescence and photoacoustic imaging agent for in vivo cancer imaging. In another example of surface functionalized CDs, Zhu’s group functionalized porphyrin-based nitrogen-rich CDs with cetuximab for precisely targeting cancer cells with overexpression of epidermal growth factor receptor, and thus enhance the photoacoustic signals in tumors [190]. The resulting NIR-CDs could significantly enhance PA amplitude signals and maintain a strong signal for 12 h in the mice bearing MDA-MB-231 breast cancer, which provides a long-term and accurate guidance for efficient photodynamic therapy for breast cancer. This is a particularly interesting example of multimodal imaging using CDs, where NIR absorbing/emission was exploited to combine fluorescence, two-photon and photoacoustic in a unique nanoplatform. However, a fine tuning of the absorption and emission wavelengths of CDs is still challenging, and there are no reliable in silico models enabling the right choice of precursors for their rational design as function of the specific application.

#### 2.3.3. Image-Guided Photothermal/Photodynamic Therapy (IG-PTT/PDT)

Combining targeted drug delivery and phototherapy (PTT) is a critical application that can be improved using CDs. CDs can act as multimodal nanoplatforms to simultaneously deliver drugs inside tumors, develop local hyperthermia or ROS by NIR stimulation and be monitored in real-time, making them eligible for image-guided ablation of solid tumors (Figure 1).

PTT can be used to thermally ablate cancer cells through photothermal agents that controllably convert absorbed NIR light into heat in targeted tumor area [185,191]. Ge et al. have developed red-emissive CDs with absorption region within the range 400–750 nm by solvothermal decomposition of polythiophene phenylpropionic acid. These CDs showed strong photoacoustic response under NIR light irradiation and high photothermal conversion efficiency (*η* ≈ 38.5%). These unique properties enabled the CDs to act as multifunctional fluorescent, photoacoustic, and thermal theranostic agents for simultaneous diagnosis and cancer therapy. Qu’s group have developed supra-CDs by solvothermal decomposition of citric acid and exhibiting high absorption in the NIR region and good NIR photothermal conversion performance [189]. Supra-CDs were explored as a photothermal agent for photothermal therapy (PTT) combined with photoacoustic imaging of cancer cells. As a result, in vivo tumor PTT is realized under 655 nm laser irradiation via both intratumor and intravenous injecting supra-CDs. In vivo PA imaging revealed that supra-CDs can accumulate in the tumor tissue via the blood circulation after intravenous injection, and the lives of mice were prolonged due to the tumor growth inhibition after PTT. Another commonly utilized carbon source for the synthesis of NIR-responsive CDs is NIR-absorbing dyes. Liu and colleagues demonstrated that using soybean milk and methylene blue as carbon sources is possible to obtain MB-containing CDs by microwave-assisted solvothermal decomposition methods. MB moieties were completely retained at the CDs surface, thus they could be further applied for photoacoustic/fluorescence dual-mode imaging-guided PTT ablation of tumors [192]. N-doped CDs surface passivated with a bifunctional amino-PEG_2000_-biotin (CDs-PEG-BT) have been demonstrated another valid strategy to obtain optimal optical properties, good biocompatibility, targeting capability and high drug loading (28.4%). CDs-PEG-BT show effective red fluorescence and photothermal conversion for image-guided photothermal therapy combined with the triggered release of irinotecan, as demonstrated by in vitro and 3D ex vivo characterization on breast cancer models [21].

Photodynamic therapy (PDT) is based on the energy transfer of photoactivable components towards photosensitizers who generate reactive oxygen species (ROS) via oxygen excitation in situ. Photosensitizers provide ROS by transferring photon energy to molecular oxygen, thus generating singlet oxygen species that mediate beneficial and localized cytotoxic effects in the target tissues that have been exposed to light [193]. These photoinduced damages can be exploited to selectively kill cancer cells in vivo in a targeted way. PDT can be easily achieved using fluorescent CDs possessing suitable photostability as shown elsewhere. In particular, the strategy consists of the overlapping of the emission band of CDs with absorption one of the photosensitizers, thus reaching highly efficient energy transfer from the CDs to the photosensitizer moiety. Choi and co-workers proposed folic acid functionalized CDs as carriers of zinc phthalocyanine, used as photosensitizer, to achieve combined bioimaging and targeted photodynamic therapy of tumors (Table 1) [138]. Folic acid was conjugated to PEG-passivated CDs to give rise to targeted delivery of the photosensitizer (loaded via π–π stacking interactions) to FA-positive cancer cells. They exhibited excellent targeted delivery of the PS, leading to simultaneous imaging and significant targeted photodynamic therapy after irradiation in vitro and in vivo. Very recently, Cai’s groups have developed sulfur-doped CDs, via a hydrothermal process using polythiophene, with high yield of singlet oxygen to improve the PDT efficacy in clinical practice for the treatment of oral squamous cell carcinoma [167]. Under light irradiation, these CDs acted as a more effective nano-weapon for anticancer therapy compared with traditional photosensitizers such as 5-aminolevulinic acid. The high therapeutic efficiency of the nanostructure was speculated to be realized by generating high rate of ^1^O_2_, inducing acute stress response and Ca^2+^ influx, and thereafter the overexpression of caspase-3 and Bax proteins as well as the downregulation of Bcl-2 protein. Chen et al. have prepared electrostatic complexes between porphyrin derivatives and CDs obtaining efficient FRET (45%), thus displaying excellent two-photon excitation in PDT [194]. These functionalized CDs were able to produce a higher amount of singlet oxygen under two-photon excitation at 700 nm fs laser if compared to the parent virgin porphyrin. This nanosystem is a potential platform for in vivo cancer eradication by means of PDT under the guidance of fluorescence and photoacoustic imaging.

Some NIR-responsive CDs display synergistic photothermal and photodynamic effects and can be employed as multimodal theranostic agents for tumor ablation in vivo [187]. For example, Shen et al. have obtained copper-doped nitrogen-rich CDs, which can both enhance the formation of ROS and production of heating under NIR irradiation at low power density. Besides, they can be used as photoacoustic agents for image-guided phototherapy (Table 1) [139]. It might be mentioned that there are still few examples of NIR-responsive CDs with these features, although there is great attention worldwide.

### 2.4. Graphene Oxide (GO)

Graphene oxide (GO) is one of the water dispersible forms of graphene, that is a single 2D sheet of atom having sp^2^ hybridized carbon atoms regularly arranged in a honeycomb like lattice (Figure 2) [195]. In this lattice structure, each atom is in the same plane via a covalent carbon–carbon bond, as the interlayers are arranged through Van del Waals interactions. The typical aromatic structure of graphene is interrupted in GO sheets owing to the presence of functional groups such as hydroxyl, carboxyl, amide and epoxy groups which have sp^3^ hybridization, and imply distortions of the plane along all 2D directions. Apart from the higher polarity of GO if compared with the virgin graphene, which provides high tendency to disperse well in aqueous media, it is highly desired in the field of theranostics since it is biocompatible, biodegradable, has huge surface area (2640 m^2^ g^−1^), high aspect ratio, high thermal conductivity (i.e., 5000 W m^−1^ K^−1^), better colloidal stability, suitable photothermal conversion in the NIR region and good capability of traversing the plasma membrane [196,197,198].

Remarkable features of GO are mainly due to its chemical modification to give rise to nanocomposite materials with a combination of properties owing to various entities such as polymers and metal nanoparticles (i.e., GNPs or SPIONs) [15,199,200,201]. In fact, on one hand, the functionalization of GO with polar polymers usually avoid the natural tendency of naked GO to aggregate in physiological media due to adsorption of proteins and salting out phenomena [25,202,203]. Polymers also reduce its toxicity and hemolytic effect [204,205]. Most of the surface functionalized GO nano-sheets include poly(ethylene glycol)-GO derivatives, consisting of GO sheets of 20–500 nm length and 1 nm thickness coupled via amidic bond with amino-poly(ethylene glycol) chains at the perimeter. Indeed, GO carries show several reactive carboxylic functions at the perimeter which can be further functionalized with polymers bearing amines or alcohols giving rise to controlled GO-polymer architectures endowed with higher stability in aqueous environment and better biocompatibility if compared to the parent virgin GO sheets [198,206,207,208]. However, these strategies do not completely avoid self-aggregation of GO sheets by π–π stacking interactions, and serious issues of stability in physiological media can occur over time [209,210,211]. On the other hand, the functionalization of the GO’s faces, together with perimetral PEGylation, circumvents this tendency since the steric hindrance due to the presence of colloids which completely cover the GO surface. In addition, even if simple PEGylation of GO has promised to improve the biological performance of GO at the bio-nano interface, there are doubts that surface passivation of GO with PEG chains only at the perimeter elicits less dramatic immune responses that their pristine counterparts.

Luo et al. have indeed demonstrated that PEGylated nano-GO sheets trigger massive cytokine responses in peritoneal macrophages, despite not being internalized. They also showed that GO preferentially adsorbs onto cell membranes, thereby amplifying interactions with stimulatory surface receptors and provoking cytokine secretion by enhancing integrin β_8_-related signaling pathways [206]. PEGylation of the GO’s faces by means of epoxy ring opening reactions with heterobifunctional amine-PEG is uncommon, but much more effective than simple coupling with carboxylic acids at the perimeter. For instance, using this approach stable ultrasmall nanosheets of 30 nm length (GO-PEG-Fol) bearing folic acid both on the planar faces and at the perimeter has been obtained [25]. GO-PEG-Fol consisted of a nano-GO sheet highly functionalized with folic acid-terminated PEG2000 chains through amidic coupling and azide-alkyne click cycloaddition. The GO-PEG-Fol incorporated a high amount of doxorubicin (drug loading > 33%) and behaves as NIR-light-activated heater capable of triggering sudden doxorubicin delivery inside cancer cells and localized hyperthermia, thus provoking efficient breast cancer death. The cytotoxic effect was found to be selective for breast cancer cells, the IC_50_ being up to 12 times lower than that observed for healthy fibroblasts. Other polymers has been employed to improve colloidal stability of GO, its biocompatibility and to confer thermos/stimuli responsiveness (i.e., poly(N-isopropylacrylamide)—PNIPAM) [37,202,203,212,213,214,215,216]. For example, covalently functionalized graphene sheets were prepared by grafting a well-defined thermo-responsive poly(*N*-isopropylacrylamide) (PNIPAM) via click chemistry, thus loading by π–π stacking interactions a water-insoluble anticancer drug, camptothecin (CPT), with a superior loading capacity of 15.6 wt % [217]. Kakran et al. pursued their studies in the development of multifunctional GO sheets carrying Tween 80, poly(ethylene glycol)-*block*-poly(propylene glycol)-*block*-poly(ethylene glycol) moieties and maltodextrin so as to load a high amount of poorly soluble anticancer drugs such as ellagic acid by π–π stacking adsorption [218]. Using this approach, they demonstrated that a pH-dependent drug release occurred, suggesting also that GO did not hamper the scavenger properties of the payload.

On the other hand, metal/GO nanocomposites provide the proper combination of magnet [219,220,221], MRI/CT imaging [218], drug delivery and photothermal [15,222] properties that make them powerful all-in-one tools useful in advanced cancer theranostic applications. These characteristics are highly desired in cancer theranostics for the development of efficient smart agents for precision cancer therapy applications.

#### 2.4.1. Biocompatibility of Graphene Oxide: An Open Debate!

Graphene oxide-based nanosystems are a class of biomaterial amenable of many functionalizations, since GO nanosheets have reactive polar surface groups (e.g., hydroxy, epoxy and carboxy functions), which make them available for several conjugation strategies. The potential combination of many biological and physicochemical properties due to the chemical versatility of this nanomaterial have attracted unprecedented attention in theranostics. However, whether GO and parent compounds are cytocompatible, or they may elicit a toxic effect both at cellular and tissue levels, remains open to debate.

Many works have shown that GO-based nanocarriers do not exhibit cytotoxicity, and thus they are commonly considered as safe materials. Recently, researchers have made efforts to systematically study the GO cytotoxicity both in vitro and in vivo [223]. They tested nano-GO (100–200 nm) and compared the obtained data with those obtained after the exposure to equivalent amount of micro-sized GO. They noticed that as nano-GO did not provoke significant cytotoxicity on cells accompanied with negligible hemolysis, micro-sized GO sheets exhibited higher hemolysis and appreciable cytotoxic effects in vitro (Figure 2). These effects remarkably decreased after surface conjugation of GO with biocompatible polymers, suggesting that the lower tendency of GO to form aggregates is the key factor to explain cytotoxic phenomena that occurred at the material–cell interface. Hence, it was deduced that surface functionalization of GO can lead to changes in its physicochemical properties, thereby impinging on the cytotoxic behavior. Interestingly, Zhang et al. showed that, compared with other carbon nanomaterials, GO exhibits long blood circulation time (half-time 5.3 ± 1.2 h), and low uptake in the reticuloendothelial system [224]. No hemolysis and pathological signs can be observed in examined organs when mice are exposed up to a dose of 1 mg kg^−1^ body weight for 14 days. Besides, even if a lot of in vitro effects of GO have been reported using 2D cultures of fibroblasts [225], endothelial cells [226] and neuronal cells [227], there are no reliable studies on complex 3D organoids mimicking physiological conditions.

On the contrary, Wang et al. suggest that when GO is explored for in vivo applications in humans, its biocompatibility should be carefully considered because it can exhibit dose-dependent toxicity to cells and animals, such as inducing cell apoptosis and lung granuloma formation [228]. In addition, GO may not be eliminated by the physiological renal clearance since it is normally greater than the renal cut-off (< 5 nm). However, there are limited studies focused on the biocompatibility of GO in living cells and animals, and further efforts should be made to corroborate existing data giving rise to a definitive verdict on the possible safe use of GO in medicine. Some authors have highlighted a possible disruptive cell-GO interaction characterized by strong distortion of the cellular membrane owing to adsorption mechanisms and hard frictions due to the very high mechanical performance of GO nanosheets [229,230]. GO not only can act as a molecular scalpel damaging cell membranes, but also can compromise cytoskeleton in J774A.1 macrophages and A549 lung cancer cells at sub-lethal concentrations [230]. Besides, mechanistic investigation suggests that interactions of GO–integrin occurred on the plasma membrane and, consequently, activated the integrin–FAK–Rho–ROCK pathway and suppressed the expression of integrin, resulting in a compromised cell membrane and cytoskeleton. The lack of convincing data on the GO biocompatibility explains well why, despite hundreds of published papers over a short period, only a few patents have been filed in the field of medicine.

In recent years, the hypothesis of GO degradation upon in vivo administration has shed light on the possible fate of GO after biodistribution in the human body, thus spicing up the debate on the rule of GO in real world medical applications. Currently, research examining the biodegradability of GO sheets is limited to a few studies [231]. This thesis starts with an in vitro study to establish the degradative reactions that occur in GO sheets under a defined oxidative influence. The author then describes the interrogation of the biodegradability of GO sheets in the spleen and the brain following intravenous and intranasal exposures, respectively, in C57BL/6 mice [232]. Even if graphene oxide was assumed to be persistent, subsequent works evidenced that peroxidases can trigger degradation of GO both in vitro and in vivo [233]. Therefore, more research should be carried out to assess biodegradation and bioelimination processes of GO in vivo in order to ensure the safety of this nanomaterial in biomedical applications.

#### 2.4.2. Biosensing

Graphene oxide has been used to design advanced nanomaterials for biosensing applications because of the possibility to adsorb both electron donor and electron acceptor dyes along the surface plane, thus making GO a suitable slab for efficient long-range photo-quenching. Indeed, Resonance Energy Transfer (RET) is a mechanism of energy transfer between a donor chromophore transfers energy to an acceptor through non-radiative dipole–dipole coupling, which requires spatial proximity. Hence, GO can be assumed as a superior acceptor for DNA-containing fluorescent probes in RET. The bases of this mechanism is that these probes can be caught by the GO surface by π–π stacking, which leads to fluorescence quenching by RET and then, after the DNA probes bind to the target sequence, fluorescence is switched on due to separation of the DNA from the GO surface in situ [234].

Very promising applications of functionalized GO probes reported so far include their use in protein and DNA sensing applications [235,236]. Wang and co-workers have explored GO nanocomplexes for intracellular monitoring and in situ molecular probing abilities in living cells. In particular, they have designed an aptamer-carboxyfluorescein supramolecular GO nanosheet to demonstrate that real-time cellular target monitoring can be successfully realized using these nanoplatforms in living cells. This is an example of reversible physical surface functionalization of GO that exploits different optical properties of supramolecular assemblies to gain stable signals useful for sensing applications. Here, GO was used as molecular sensing plane and aptamers as quenched molecular probes that, after recognition of adenosine triphosphate (ATP) by ATP-ATP aptamer recognition domains, provide fluorescence signals acting as an efficient probe for ATP detection. Besides, they demonstrated that GO is able to protect and deliver aptamers in living cells, thus suggesting a potential key role of GO as an excellent vehicle to transport gene into cells, protecting the loaded gene from enzymatic cleavage and enabling in situ molecular probing in living cells [236]. He et al. used multi-color fluorescent probes to detect specific target sequences and rapidly attained highly selective multiplex sequence-specific DNA detection in complex environmental solutions. They also used this technology to effectively distinguish sequences with single-base errors, suggesting a great potential of GO for cancer gene detection. Furthermore, the availability of large planar surfaces of GO makes it possible to detect multiple molecular targets in the same solution [237]. Using a similar approach, Luo et al. developed an efficient strategy to enhance chemiluminescence biosensor for ultrasensitive sequence specific single stranded DNA detection [238]. They combined the ability of GO of inhibiting the peroxidatic activity of a horseradish peroxidase-mimicking DNAzyme with the peculiar interactions usually occurred between GO and single stranded DNA. In this particular work, human immunodeficiency oligonucleotide sequence associated with HMDNAzyme (a catalyst able to produce fluorescence in the presence of luminol and hydrogen peroxide) was used as a probe that, after proper adsorption onto the GO plane by means of π–π interactions, underwent fluorescence quenching. Thus, the recognition of the complementary target DNA provoked the formation of the double stranded DNA with the probe and its release from the GO surface, yielding to a significant chemiluminescence increase owing to the transformation of luminol in solution (dequenching). This supramolecular nanosystem is assumed to be a promising candidate for sequence specific detection not only of DNA, but also for other biomolecules such as proteins and RNA. A similar supramolecular nanosystem can be designed to act as a sensor of biomarkers for the diagnosis of cancers as well as for the monitoring of the physiopathology of tumors during the treatment in view of personalized and precise anticancer approaches.

In another study, GO has been used as peptide biosensor exploiting the GO complexing abilities toward a pyrene-RGD conjugate [239]. Cooperative π–π interactions between pyrene and GO surface give rise to supramolecular nanosystems characterized by quenched fluorescence and the ability of releasing the pyrene moiety (endowed with inherent green emission bands) due to competitive binding of RGD with integrin ανβ3 overexpressed in blood vessels nearby tumors. The increment of fluorescence can be used to track cancer cells’ adhesion and proliferation behaviors and as biosensor for real-time specific biomarker detection on the cell surface.

Utilizing GO as a biosensor tool in liquid biopsy of cancer has effectively improved the sensitivity and specificity of biosensors for cancer detection. Mei Heb and co-workers have modified GO nanosheets with a layer of polydopamine and used protein G to immobilize antibodies on GO for exosome capture useful for liquid biopsy applications [240].

#### 2.4.3. Image-Guided Photothermal Therapy (PTT) Using Graphene Oxide

Various reports proposed elsewhere show that GO can be diligently functionalized to be employed not only for the selective delivery of anticancer agents into tumors, but also for mini-invasive photo-induced therapy of solid tumors such as phototherapy (PTT) and photodynamic therapy (PDT). Combinational strategies applying PTT and PDT at once have been extensively studied, while simultaneously exciting with a single laser in the biologically transparent window (NIR wavelength) remains challenging. Ideally, GO can act as a photosensitizer aimed at achieving cell death through the generation of reactive oxygen species (ROS) inside cancer cells. This will result in localized oxidative photodamage, consisting in the oxidation of cellular bio-elements, including nucleic acids, lipids, and proteins, thus leading to selective cytotoxicity mainly due to severe alterations in cell signaling cascades and gene expression regulation [129,241,242]. In a very interesting work, Liu et al. have developed PEGylated nano-GO co-loaded with photosensitizers and a two-photon compound as a theranostic nanomedicine against cancer. A two-photon compound was employed to convert near-infrared laser into visible light to excite the photosensitizer thus achieving deeper therapeutic depth (Table 1). Besides, the two-photon compound was activated quickly after releasing from the carrier in vivo, providing imaging abilities [140]. They showed that this combined approach attacked 4T1 murine breast cancer cells and induced apoptosis. Another possible therapeutic approach is due to the development of localized hyperthermia (42–42 °C) by transforming near infrared (NIR) light into heat, namely by photothermal therapy (PTT), which afford to selective cancer cell death or ablation (T > 46 °C). Various studies showed that GO nanosheets can absorb 700–810 nm diode laser light so as to release heat inside cells, yielding to cancer cell hyperthermia and preferentially killing cancer cells instead of healthy ones [203,220,243,244,245]. For instance, GO has been functionalized by self-assembling with inulin prodrugs carrying doxorubicin covalently linked by pH-sensitive citraconylamide spacers [202]. Doxorubicin has been adsorbed onto GO surface by π–π stacking to be release both at acidic conditions mimicking tumor microenvironment and on demand after proper NIR light stimulation. A massive drug release (100%) was obtained after NIR light triggering for 300 s at suitable power density, and higher cytotoxic effects has been observed for the nanosystems bearing biotin moieties as side chains, since biotin receptors on cancer cell membranes provide higher penetration efficiency if compared with the normal nanosystem. In another study, Tran et al. have proposed a chemo-photothermal synergistic therapy based on the use of dual anticancer drug-loaded GO stabilized with poloxamer 188 for generating heat and delivering drugs to kill cancer cells under near-infrared (NIR) laser irradiation [246].

However, the application of GO in cancer theranostics is mostly precluded owing to the lack of specific contrast properties. As a consequence, many attempts have been made to exploit GO/metal and GO/CDs nanocomposites for combined multimodal diagnosis and photothermal therapy. In principle, this peculiar theranostics application implies high selectivity and efficacy through image-guided ablation techniques (e.g., MRI/FL-guided phototherapy) [247,248,249]. For example, SPIONs/GO nanocomposites were prepared by covalent amide bonds through aminodextran-functionalized SPIONs and the carboxylic acid of GO at the surface (Table 1). This nanocomposite was stable under physiological conditions and it was found to be useful as T2-weighted MRI agent since SPIONs aggregation at the GO surface ensures enhanced T2 relaxivity and suitable MRI abilities [141]. Prussian Blue staining analysis indicates that the nanocomposites can be internalized efficiently by HeLa cells and can be potentially used in MRI-guided PTT applications. In another attempt GO has been exploited to design nanocomposites for phototherapy combined with MRI and photoacoustic tomography (PA). Here, SPIONs were seeded on the GO surface by nanoprecipitation and PEG-grafted poly (maleic anhydride-alt-1-octadecene) (C18PMH–PEG) was synthesized and used as coating agent by self-assembling, obtaining a nanocomposite with excellent physiological stability, strong NIR optical absorbance, and superparamagnetic properties (Table 1). Using this theranostic nanoprobe, in vivo triple modal fluorescence, photoacoustic, and magnetic resonance imaging were carried out, uncovering high passive tumor targeting, which is further used for effective photothermal ablation of tumors in mice under the guidance of imaging [142]. Gold nanoparticles/GO nanocomposites has been explored as theranostic agents useful in positron emission tomography (PET) imaging as well [15,205,218]. In a particular work, GO was functionalized with a six-arm amino-PEG carrying an active targeting ligand (TRC105, a monoclonal antibody that binds CD105), leading to significantly improved tumor uptake of functionalized GO (specific for the neovasculature with little extravasation) and warranting future development of these GO conjugates for cancer-targeted PET/CT-guided drug delivery and PTT [250]. In another study, Sheng et al. have developed BSA-functionalized GO by a simple in situ reduction of GO, thus restoring sp^2^ hybridization of graphene comb-like structures and high NIR photothermal conversion. BSA provided high stability under physiological conditions and high cytocompatibility. In particular, they showed that these nanomaterials had no cytotoxic effect on the MCF-7 cell lines at concentrations up to 40 mg mL^−1^. The resulting nanosized BSA-GO conjugate displayed PA and ultrasonic dual-imaging potential useful for image-guided PTT in living mice [251]. In another approach, Miao and co-workers have developed image-guided synergistic photothermal antitumor effect of photo-responsive NIR imaging agent, namely indocyanine green (ICG), by loading it onto hyaluronic acid-anchored GO nanosheets [252]. Surface modification of GO with ICG improved the photostability upon NIR irradiation, while hyaluronic acid pendants provided greater cellular delivery of ICG and photothermal tumor ablation upon laser irradiation in CD44-positive KB cells. They also demonstrated the ability of this nanocomposite to image KB tumor-bearing mice in vivo, implying selective PTT under the guidance of photoacoustic imaging. Using a similar approach, Seng et al. have designed a GO-based nanosystem carrying ICG for fluorescence imaging-guided synergistic phototherapy of drug-resistant osteosarcoma. This work proposed a drug delivery system based on near-infrared imaging and multifunctional GO, which can target mitochondria showing synergistic phototherapy with preferential accumulation in tumors [247]. They functionalized GO via amide coupling with branched polyethylenimine-modified amino-PEG and a mitochondria-targeting ligand ((4-carboxybutyl) triphenyl phosphonium bromide) to promote mitochondrial accumulation after cellular internalization.

## 3. Summary and Future Perspectives

Various kinds of metal- and carbon-based nanoparticles or hybrid nanomaterials have been designed and developed, and among these are some expected to provide significant improvements in the field of precision cancer therapies, diagnosis and monitoring of solid tumors. In this review, we systematically disclose the potential applications of the most advanced gold-, iron oxide superparamagnetic-, graphene oxide- and carbon nanodots-based theranostic agents useful in precision and personalized cancer therapy as well as in bioimaging and biosensing applications. Recent works have shown that well-designed nanosystems with tunable interfacial, physical-chemical and optical features could be obtained in order to display multimodal imaging and selective non-invasive eradication of tumors. There is also a growing interest in developing photoactivatable theranostic nanosystems for even-smarter cancer therapy and imaging. Each type of nanosystem seems to have some characteristic advantage, but it might be noticed that better results could be obtained by designing multimodal nanohybrid “machines” capable in a single platform of imaging, monitoring and sensing tumors (especially tumor microenvironment) while treating cancer cells. This promises to predict therapeutic responses together with a selective cancer cells eradication under continuous monitoring. Overall, hybrid nanotheranostics represent the future of personalized cancer treatments and require more in-depth knowledge of basic information about tissue/nanomaterial interactions to develop safe nanomedicines. Besides, further efforts should be made to design smart nanomaterials with well-established architectures (i.e., size, composition and surface features) using highly tunable, industrially scalable and cost-effective synthetic routes to overcome the existing regulatory barriers that have limited their translation into the clinic.
